# High school English-as-a-foreign-language teachers’ emotional labor and job satisfaction: A latent profile analytical approach

**DOI:** 10.3389/fpsyg.2022.950229

**Published:** 2022-08-02

**Authors:** Shenhai Zhu, Maojie Zhou

**Affiliations:** ^1^College for Foreign Studies, Guangxi Normal University, Guilin, China; ^2^School of Foreign Language and Literature, Xiangsihu College of Guangxi University for Nationalities, Nanning, China

**Keywords:** emotional labor, job satisfaction, latent profile analysis, high school EFL teachers, classification of teachers

## Abstract

Previous studies have primarily used variable-centered approaches to explore correlations between English-as-a-foreign-language (EFL) teachers’ emotional labor and outcome variables. A fundamental but unresolved question is whether teachers employ multiple emotional labor strategies in the workplace. This study used the latent profile analysis (LPA) to explore the profiles of EFL teachers’ emotional labor and the relationship between the profiles and job satisfaction based on a questionnaire survey of 365 high school EFL teachers in China. The results indicated the existence of three emotional labor profiles—agreeable, neutral and emotional—that were characterized by different combinations of surface acting (SA), deep acting (DA), and expression of naturally felt emotion (ENFE). The profiles of EFL teachers who predominantly relied on ENFE and DA had the most adaptive patterns of job satisfaction, whereas the profiles of teachers who reported higher levels of SA, regardless the level of ENFE and DA, experienced lower levels of job satisfaction. These findings provide a person-centered methodological data support for exploring the relationship between emotional labor and EFL teachers’ job satisfaction.

## Introduction

Over the past 40 years, the basic English education system in China has changed tremendously due to the country’s reform and opening up.^1^ The *General Senior High School English Curriculum Standards (Version 2017)*, introduced by the Ministry of Education of the People’s Republic of China in 2018, marked a new stage in the development of the high school English language curriculum. However, in recent years, English-as-a-foreign-language (EFL) teacher attrition has increased as Chinese high school education has become more advanced, which has led to problems of optimizing the teaching structure in schools (Zhang, 2011). An area of research that may shed light on the pressing problem of teacher attrition is emotional labor (Acheson et al., 2016). To advance foreign language (FL) talent in the new era, EFL teachers are expected to provide high-quality teaching. Besides the heavy burden of test-based education (Cheng and Wang, 2004), the conflicts between a neoliberal educational culture, teacher accountability, government language policies, an English instrumentalist orientation, teaching to the test, administrators’ expectations, parental demands, and EFL teachers’ views of language, education, the curriculum, and critical thinking (Benesch, 2020) have all led EFL teachers to expend more emotional labor on their work (Yin, 2015).

However, research on language teachers’ emotions is relatively rare compared to research focusing on language learners’ emotions (Miller and Gkonou, 2018; Greenier et al., 2021). While the few relevant studies have been based on different theoretical constructs, they have consistently called for more empirical work on how emotions are formed, unfold in teachers’ lives, and affect teachers’ personal and professional development and well-being to provide a more nuanced understanding of ways to tackle these issues (Miller and Gkonou, 2018; Dewaele and Wu, 2021; Li and Liu, 2021; Shen, 2022). In view of this situation, it is necessary to study the emotional labor of high school EFL teachers and its influencing mechanisms. Job satisfaction is one of the frequently studied outcomes related to the teaching profession (Al’Abri et al., 2022). It is an important indicator of teachers’ well-being, and it is also an important variable influencing attrition (Price, 2001; Antón, 2009). This suggests that examining the relationship between EFL teachers’ emotional labor and job satisfaction may have positive implications for teachers’ professional development.

Current empirical research on the relationship between teachers’ emotional labor strategies and job satisfaction has yielded a variety of findings (Kinmana et al., 2011; Li and Wang, 2014). In general, most studies on teachers’ emotional labor have used variable-centered approaches (Craig and Smith, 2000), using one-to-one mapping between each emotional labor strategy and job satisfaction. However, this mapping may be difficult to generalize to real-life situations (Cheung et al., 2018), and the results fail to adequately consider the overall tendencies and types of differences in teachers’ emotional labor. Therefore, more systematic quantitative research is needed to measure EFL teachers’ emotional labor strategies.

In recent years, studies using person-centered approaches to explore teachers’ emotional labor have proved their usefulness (e.g., Gabriel et al., 2015; Fouquereau et al., 2018). However, few studies have used such approaches. Exploring how teachers who employ different combinations of emotional labor strategies and their relationships with various outcome variables is relevant not only for reconciling the contradictions and refining theoretical knowledge in the literature, but also for securing the necessary resources to promote and sustain EFL teachers’ well-being and job satisfaction (Burić et al., 2020).

Given the shortcomings of related research and the importance of professional psychological research for EFL teachers, we designed this study to examine the profiles of high school EFL teachers’ emotional labor using a person-centered approach. We considered it a top priority to apply the latent profile analysis (LPA) and to identify distinct types of emotional labor among this group of teachers. We then linked teachers’ different potential profiles of emotional labor to job satisfaction to explore the correlations between them. Overall, we aimed to clarify the different emotional labor profiles of EFL teachers in the context of Chinese teacher education and to clarify the usefulness of these profiles in describing the current state of teachers’ job satisfaction.

## Literature review

### English-as-a-foreign-language teachers’ emotional labor

The concept of emotional labor was first introduced by Hochschild (1983). In a study of Western Airlines flight attendants, she found that they engaged not only in physical and mental labor but also in emotional labor. She defined emotional labor from a mimetic perspective as “the management of feeling to create a publicly observable facial and bodily display” (Hochschild, 1983, p. 7). Later, based on a review of different conceptualizations of emotional labor, Grandey (2000) identified similarities in the underlying strategies that individuals used to regulate their emotional expressions at work. Thus, emotional labor has been defined as “the process of regulating both feelings and expressions for the organization goals in psychology” (Grandey, 2000, p. 97).

Teaching satisfies all Hochschild (1983) three criteria for jobs that require emotional labor: (1) face-to-face contact is required between teachers and others, especially students; (2) teachers experience certain emotional states (e.g., joy, fear, excitement, or anxiety) in the presence of students or those around them; and (3) teachers’ emotional labor in the classroom or school setting is subject to some degree of extrinsic control, which usually derives from cultural expectations or professional standards (Winograd, 2003). Furthermore, teachers take for granted that they should follow specific display rules in their classrooms, including showing or adjusting to positive emotions and suppressing negative ones (Zembylas, 2003; Williams-Johnson et al., 2008; Scott and Sutton, 2009). This has led to a consensus that teaching is a form of emotional labor (Yin et al., 2017), which, since the 1990s, has led to an increasing number of researchers focusing on the importance of teachers’ emotions in teaching and learning (Li and Liu, 2021). Recent findings have confirmed that teachers’ emotional behaviors affect students’ motivation (Pishghadam et al., 2021a), willingness to listen (Pishghadam et al., 2021b), and emotional experiences in the classroom (Derakhshan et al., 2021), among other factors. Emotional labor has become an essential element in research related to teachers’ emotions (Wang et al., 2021). However, because teachers’ emotional expressions vary in different educational and cultural settings (Krone and Morgan, 2000), the emotional labor of high school EFL teachers, which differs from that of teachers of other subjects, requires further research.

To promote effective teaching and maintain positive relationships with learners, EFL teachers need to engage in emotional labor in their relationships with students, parents, and institutions while regulating their own emotional states (Cowie, 2011; King, 2015). In such situations, teachers may not develop adequate strategies for regulating their emotions and may become exhausted (Chang, 2009). Unfortunately, research on EFL teachers’ emotional labor has progressed only slowly (Li and Liu, 2021) and, to date, few studies have delved into the emotions associated with, or how they relate to, English language teaching (ELT) in different sociocultural contexts. In particular, high school EFL teachers are torn between short-term teaching tasks and long-term English educational goals in the Gaokao^2^ -oriented school environment. When they are forced to readjust their teaching practices and beliefs, they may feel emotionally stressed (Assaf, 2008). Also, high school EFL teachers not only need to regularly use emotional labor to improve teacher–student relationships and reduce students’ anxiety about language learning, but must also cope with and overcome public criticism and the ensuing loss of public confidence in their ability to provide high-quality language instruction to their students (Yan, 2014)—a process that is inevitably fraught with emotion.

In general, research on EFL teachers’ emotional labor started later than that on teachers’ emotional labor, but the two have developed in the same vein. Although the professional nature of foreign language teachers determines its commonality with teachers of other subjects, the fact that the profession of foreign language teachers is language and that the purpose of foreign language education is to achieve language-mediated cultural exchange also determines the uniqueness and contextual typicality of foreign language teachers. Researchers have noted that teachers within specific subject areas should receive instruction on how to deal with the emotions prevalent in their fields (Williams-Johnson et al., 2008). Teaching and learning English are embedded in an “emotional ecosystem” (Benesch, 2012), and teachers’ emotional expressions vary across educational and cultural contexts (Krone and Morgan, 2000). In particular, we have only a limited understanding of the development and expression of teachers’ emotions outside the “Western academic mainstream” (Chen, 2019). Thus, the emotional labor of EFL teachers in the Chinese sociocultural context warrants further exploration.

### English-as-a-foreign-language teachers’ emotional labor strategies

Like other subject teachers, such as math and science teachers, language teachers regulate their emotions to create appropriate expressions of emotion when handling students or their learning concerns (Golombek and Doran, 2014). Previous empirical studies (Constanti and Gibbs, 2004; Cukur, 2009; Yin, 2012; Yin et al., 2017; Benesch, 2018) verified that teachers engage in emotional labor by utilizing surface acting (SA), deep acting (DA), and expressions of naturally felt emotion (ENFE). SA refers to a strategy whereby teachers “stimulate emotions that are not actually felt or change the outward expression of felt emotion” (Cukur, 2009, p. 561). DA means “the emotion management process in which teachers try to modify their felt emotions using some cognitive techniques (e.g., distraction and self-persuasion)” to display appropriate emotions and behaviors (Yin, 2012, p. 452). In short, DA is an antecedent-centered emotion regulation, whereas SA is a reaction-centered emotion regulation (Gross, 1998; John and Gross, 2007). In contrast, the third strategy (ENFE) occurs when felt emotions are in line with emotional rules (Li and Liu, 2021).

To date, empirical research on EFL teachers’ use of emotional labor strategies has analyzed teachers’ motivations for applying these strategies, as well as the processes and outcomes of their application (e.g., Zhang and Zhu, 2008; King, 2015). To maintain a positive atmosphere, EFL teachers generally choose to display a positive and proactive attitude toward language learning to communicate their own motivation to learners rather than exposing their frustrations in the classroom. For example, they sometimes play the role of “cheerleaders” and suppress their negative emotions (King, 2015). Teachers also make supreme efforts to build personal relationships with learners by praising them or building their confidence (Acheson et al., 2016). Whereas EFL teachers’ excessive suppression of true feelings can lead to teacher burnout, their effective regulation and engagement of emotions can increase both language teachers’ efficacy and their own teaching motivation (Kim and Kim, 2018). These studies have confirmed that the use of emotional labor strategies can be both positive and negative for FL teachers, depending on which strategy is more prominent. They also confirmed the key role of emotional labor strategies in EFL teaching processes (Greenier et al., 2021).

The few studies that have focused on high school FL teachers have primarily analyzed the emotional dilemmas they encounter and the strategies they utilize to manage their emotions. However, there are no fixed conclusions about the specific dimensions of their strategies (Li and Liu, 2021). Focusing on Korean secondary English teachers’ conflicting stories about ESA returnees in their classrooms, Song (2016) illustrated the leading role of emotions in their negotiation of conflicting stories and self-transformation. The study pointed out a critical but overlooked area of language teachers’ identities: how emotions influence language, their construction and presentation of identity, and their practices in the classroom. Acheson et al. (2016) stated that we should be aware of the importance of teachers’ emotional labor in FL pedagogy and its potential role in teacher attrition. Their study confirmed that all three of Hochschild (1983) types of emotional labor (emotional consonance, DA, and SA) were discussed by high school FL teachers, and they explored the emotional labor of five teachers in rural American high school FL classrooms. The least frequently mentioned of the three types of emotional labor was emotional concordance, possibly due to its unconscious nature—that is, FL teachers engage in caring about their students without deliberate effort and thus may not explicitly articulate all the ways in which this type of emotional labor manifests itself in their daily professional lives.

Most of the existing research on EFL teachers’ emotional labor strategies has focused on college teachers or whole groups of teachers, with less attention paid to individual high school EFL teachers. High school teachers prioritize student achievement more than teachers at other grade levels (Marston et al., 2005; Jennings and Greenberg, 2009). In the classroom, it is important for teachers not only to regulate students’ negative emotions but also to promote positive emotions in their learning (Hargreaves, 2000; Jennings and Greenberg, 2009). The high school EFL teachers in extant studies came from different sociocultural backgrounds, such as the United States (US) and Korea. Given the cultural contextual differences in the use of teachers’ emotional labor strategies and the emotional rules that govern teachers’ emotional labor (Zembylas, 2005), it is worth exploring high school EFL teachers’ emotional labor strategies and their influences in Chinese sociocultural settings.

Considering the professional development and burnout risks of high school EFL teachers, their emotional labor should be considered. Existing empirical studies have confirmed the key role that emotional labor strategies play in EFL teaching processes (Greenier et al., 2021). In reviewing previous studies, we found that most of the previous research on Chinese teachers has focused on variable-centered approaches to describe the extent to which teachers use each emotional labor strategy and reveal the correlation between each strategy and other variables (e.g., Cheung et al., 2011; Han et al., 2020, 2021). Some findings have tentatively confirmed the coexistence of different emotional labor strategies among teachers (Sutton, 2004; Zhang and Zhu, 2008). Furthermore, although individuals may use two or more emotion regulation strategies at work, their tendency to use these strategies may manifest in specific styles. Individuals’ experiences in the workplace may vary greatly depending on their personal characteristics (Judge et al., 2017), which leads to an important and unexplored question: What are the outcomes of EFL teachers using different combinations of emotional labor strategies (e.g., DA, SA, and ENFE simultaneously) in their jobs? In this study, we aimed to use LPA to determine the behavioral characteristics of teachers in their use of the three emotional labor strategies and the outcomes associated with these profiles.

### A person-centered approach to emotional labor

To our knowledge, few studies have examined EFL teachers’ emotional labor using person-centered approaches, and there is an even greater lack of research exploring the traits of teachers’ emotional labor (e.g., Cheung and Lun, 2015; Fouquereau et al., 2018; Burić et al., 2020). Compared to traditional approaches, such as regression or structural equation modeling, LPA enables a better estimation of how the patterns of use (i.e., classes) of different emotional labor strategies in the workplace relate to job satisfaction (Cheung and Lun, 2015). As discussed earlier, it is likely that EFL teachers in work settings adopt different emotional labor strategies depending on the current availability of resources; thus, LPA may be a more realistic approach for discovering different combinations of EFL teachers’ emotional labor strategies and how these “classes” relate to health and job outcomes, such as job satisfaction.

Despite the paucity of research using LPA to explore the behavior of EFL teachers in using emotional labor strategies, the studies that have been conducted in other fields can nevertheless provide some insight. For example, Gabriel et al. (2015) first used a person-centered approach to study the emotional labor characteristics of employees in the US service industry. The results confirmed the existence of five profiles: deep actors (predominantly using DA), non-actors (using low levels of both DA and SA), low actors (using neither DA nor SA), surface actors (predominantly using SA), and regulators (flexibly using DA and SA).

Cossette and Hess (2015) analyzed suppression, reappraisal, and naturally felt emotions using LPA based on a model of emotion regulation, and the results revealed four profiles: the flexible (using all three strategies), the authentic group (mainly using reappraisal and expression), the suppressors (preferentially using suppression) and the non-regulators (using all three strategies to a low degree).

A recent study by Burić et al. (2020) went a step further and identified six emotional labor profiles in a large sample of teachers based on DA and two aspects of SA (i.e., hiding feelings and faking emotions). The researchers found that among teachers who engaged in more or less emotional labor, there were “parallel” potential profiles (i.e., combinations of similar emotional labor strategies). For example, the “low regulators” and “regulators” in the study had similar patterns of emotional labor (i.e., DA was stronger than hiding feelings, which in turn was stronger than faking emotions) but differed in that regulators used these strategies more frequently than low regulators (Burić et al., 2020).

Subsequently, a study based on a sample of 262 Chinese teachers reported three emotional labor characteristics: “emotionally congruent employees” (with high levels of DA and ENFE), “display rules compliers” (with high levels of SA and ENFE), and “active actors” (with high levels of all three strategies) (Cheung and Lun, 2015). Also, the study compared job burnout and job satisfaction among the three classes and found that emotionally congruent employees tended to report relatively low levels of job burnout and elevated levels of job satisfaction, while the display rules compliers reported the highest levels of job burnout and low levels of job satisfaction.

Like the factor labeling process in exploratory factor analysis, LPA makes it possible to assign labels to groups of individuals who comprise particular classes (McLarnon et al., 2015). Our literature review revealed different labeling strategies that, for example, described the content of profiles using indicators (e.g., Gabriel et al., 2015; Burić et al., 2020) or assigned names to capture the essence of profiles (e.g., Cheung and Lun, 2015). Currently, there is no consensus regarding a final solution, and naming may relate to the included indicators and the levels within profiles or aim to capture the essence of the respective profiles (Spurk et al., 2020).

In summary, a person-centered approach can provide a new perspective for analyzing teachers’ emotional labor and the associated problems. It can encourage researchers and practitioners to focus on individuals rather than on average scores derived from groups of individuals, which may advance educational and organizational science (Zyphur, 2009). Moreover, as an emerging person-centered research approach, LPA can provide valuable information about the relationship between emotional labor profiles and job satisfaction or well-being (Cheung et al., 2018). To the best of our knowledge, there is a lack of LPA for EFL teachers’ emotional labor. To enrich the research findings in this area, we aimed to explore groups of teachers with unique emotional labor patterns in the Chinese study sample.

### Well-being outcome of teachers’ emotional labor profiles

Latent profile analysis (LPA) allowed us to test the correlations between potential profiles and the external variables of interest, thus helping to confirm the relevance, theoretical significance, and validity of the potential profile model (Wang and Hanges, 2011). Empirical research has shown that emotional labor contributes to teachers’ job satisfaction, teaching effectiveness, and psychological well-being (Wróbel, 2013; Yin et al., 2013; Yilmaz et al., 2015). Thus, researchers have noted that the exploration of teachers’ emotional labor, particularly teachers’ regulatory behaviors, can help deepen researchers’ understanding of the correlation between emotional labor and outcomes, which is important for both teachers and their students (Horner et al., 2020).

Job satisfaction—one of the most popular well-being outcomes related to emotional labor (Grandey and Gabriel, 2015) and a specific variable of interest in this study—refers to a pleasurable or positive emotional state resulting from an individual’s job and work experience (Locke, 1976). In educational settings, Ho and Au (2006, p. 172) defined teacher’ job satisfaction as “a function of the perceived relation between what one wants from one’s job and what one perceives teaching as offering or entailing.” Individual job satisfaction is influenced by certain factors (Smith et al., 1969). The two-factor theory (Spillane, 1973) divides the factors affecting job satisfaction into intrinsic and extrinsic factors. Intrinsic factors, also known as motivational factors, relate to work, such as a sense of achievement, personal or professional growth, and promotion. Extrinsic factors are health factors, including interpersonal relationships, management policies, salaries and benefits, and similar. Studies have established that intrinsic factors play a significant role in teachers’ job satisfaction (e.g., Rice and Schneider, 1994; Scott et al., 1999). Previous research on EFL teachers’ job satisfaction has confirmed that teachers are most satisfied with the intrinsic factors of their jobs but express dissatisfaction with extrinsic factors related to school structures and policy development (e.g., Karavas, 2010).

Person-centered approaches are inherently exploratory, making it difficult to formulate specific *a priori* assumptions. However, discourse regarding the conservation of resources theory (COR; Hobfoll, 1989) and the self-regulation model (Baumeister et al., 2007) has revealed that the combined use of these emotional labor strategies relates to teachers’ job satisfaction. Based on the COR, some studies have demonstrated that emotional labor strategies play distinct roles in teachers’ job satisfaction because of differences in their effects on psychological resources. Specifically, SA was found to be significantly associated with low job satisfaction, especially the relationship between SA and extrinsic job satisfaction (EJS), which was more negative than that between SA and intrinsic job satisfaction (IJS), because it consumed more resources and contributed none (Cheung et al., 2011; Li and Wang, 2014). As with SA, DA is significantly related to the individual and organizational factors affecting teachers. Controversially, there is still debate about whether DA is beneficial in enhancing teachers’ well-being (Näring et al., 2012; Tsang et al., 2021).

Cheung and Lun (2015), using LPA, found that the adoption of DA was not associated with high levels of well-being (i.e., job satisfaction or psychological comfort) when combined with SA. In contrast, their results revealed that combining ENFE and DA may be associated with greater well-being.

Considering the complexity of the correlation between EFL teachers’ emotional labor and job satisfaction, and the inconsistency of previous research findings, it proved difficult to formulate specific hypotheses about the number and types of emotional labor profiles. However, we expected to find several types characterized by different combinations of emotional labor strategies. The empirical research outlined previously has generally used variable-centered approaches to explore teachers’ holistic emotional labor and its relationship to holistic job satisfaction, as well as constructive emotional labor and its relationship to holistic job satisfaction. However, both emotional labor and job satisfaction are multiconstruct concepts. Thus, based on the theoretical considerations and empirical results outlined previously, we expected that the profiles of EFL teachers who relied more heavily on SA (regardless of their use of DA or ENFE) would also have low levels of IJS and EJS. In contrast, we predicted that high school EFL teachers who were more prone to engage in DA and ENFE and less likely to use SA would be associated with high levels of IJS and EJS.

## Materials and methods

### Participants

The survey was conducted in March 2019. The sample consisted mainly of high school EFL teachers from the western, eastern, and central provinces of mainland China (e.g., Sichuan Province, Guangxi Zhuang Autonomous Region, Jiangsu Province, and Jiangxi Province). We used purposive sampling to distribute the questionnaires to the teachers. Before sampling, we explained the significance and purpose of the study to the interested participants, promised data anonymity and confidentiality, obtained their trust and cooperation, and sent a link to complete the questionnaire for data collection after obtaining their informed consent. Since the questionnaires for this study were distributed through an online platform, all questions were compulsory, so there were no missing values.

We collected 401 questionnaires. According to Leiner (2019), a short response time was found to be an important indicator of invalid answers. We chose to eliminate invalid questionnaires by defining the response time (less than 3 mins and more than 40 mins) and manually screening those questionnaires that had a clear pattern of responses, resulting in 365 valid questionnaires. The sample consisted of 122 males and 243 females. The highest percentage of teachers with 16 years or more of teaching experience was 37.63%, followed by 11–15 years (21.65%), 6–10 years (18.04%), 2 years or less (12.37%), and 3–5 years (10.31%), respectively. As to types of schools, 150 teachers from elite high school (41.09%), 215 teachers from ordinary high school (58.91%).

### Measures

#### Emotional labor strategies

Developed by Diefendorff et al. (2005), the Emotional Labor Scale used in this study is one of the main instruments for measuring emotional labor strategies (see Appendix A). To make the scale and questions more relevant to the teachers’ work, we adjusted the wording of some of the items. Heggestad et al. (2019) argued that it is acceptable to make minor adjustments to the wording of items, such as replacing a common word with a similar word, to improve the comprehension of the study respondents. The main changes in this study were the replacement of “customer” with “student or colleague”. For example, ‘The emotions I show customers come naturally’. was adjusted to ‘The emotions I show students or colleagues come naturally’. The scale consisted of three subscales for SA, DA, and ENFE, with the SA scale containing seven items, the DA scale containing four items, and the ENFE scale containing three items. Each item had a 5-point Likert-type scale ranging from 1 (never) to 5 (always). In the present study, internal consistency was satisfactory, with the Cronbach’s alpha coefficients of 0.90, 0.82, and 0.80 for SA, DA, and ENFE, respectively.

#### Job satisfaction

Teachers’ job satisfaction is mainly expressed as IJS and EJS. We adopted the Minnesota Satisfaction Questionnaire—Short Form developed by Weiss et al. (1967) to examine the current job satisfaction of EFL teachers (see Appendix A). This scale has been widely used and is one of a classic scale for measuring job satisfaction with good reliability. The scale contained 20 question items (the IJS subscale and the EJS subscale both contained 10 items). Respondents were asked to rate their satisfaction on a 5-point Likert-type scale ranging from 1 (very dissatisfied) to 5 (very satisfied). In this study, the Cronbach’s alpha coefficients for the subscales were 0.91 (IJS) and 0.92 (EJS), indicating that the scale also had high reliability in the present study.

### Data analysis

SPSS 21.0 and Mplus 7.4 were used to analyze the data. The descriptive statistics (means and standard deviations) and correlations were calculated using SPSS. We then conducted confirmatory factor analysis (CFA) and latent profile analysis (LPA) using Mplus. The acceptance of models was based on the following goodness-of-fit statistics: the Comparative Fit Index (CFI) and the Tucker–Lewis Index (TLI) of no less than 0.90, and the Root Mean Square Error of Approximation (RMSEA) of no more than 0.10 (Hair et al., 1998; Kline, 2005).

Since the number of latent classes was unknown and could not be directly estimated using a model, we began by specifying two latent profiles and increased the number of latent profiles until the increase in model fit no longer merited a reduction in parsimony achieved by specifying another latent class (Nylund et al., 2007). Based on the recommendations of Foti et al. (2012), we used seven main statistical indicators to determine the best-fit model: Log Likelihood (LL), Akaike Information Criterion (AIC), Bayesian Information Criterion (BIC), sample-size-adjusted BIC (SABIC), Entropy, Lo-Mendell-Rubin (LMR) test, Bootstrapped Likelihood Ratio Test (BLRT). Of these, LL, AIC, BIC, and SABIC were used as common information evaluation indicators for model comparison, and the smaller the value, the better the model fit. Entropy ranging from 0 to 1 was used as a standardized index to judge the accuracy of the model classification: the closer the value was to 1, the more accurate the classification was. We used LMR-test to compare two nested models [a *k*-category model and a (*k* − 1)-category model], and the most commonly used indexes were LMR-test and BLRT. If the LMR-test and BLRT values of the *k* model reached a significant level (*p* < 0.05), it indicated that model *k* had a greater explained variance than model *k* − 1.

## Results

### Construct validity, reliability, and correlations

A series of CFAs was conducted to test the factor structure of the scales. The emotional labor strategies scale exhibited the strongest empirical and conceptual fit. The construct validity based on the CFA indicated a good model fit (χ^2^ = 202.57, *df* = 74, RMSEA = 0.069, CFI = 0.95, TLI = 0.94).

The CFA fit indices for both IJS (χ^2^ = 164.00, *df* = 35, RMSEA = 0.092, CFI = 0.95, TLI = 0.93) and EJS (χ^2^ = 139.42, *df* = 35, RMSEA = 0.090, CFI = 0.95, TLI = 0.94) were within acceptable limits.

Table 1 reports the descriptive statistics, reliability and correlation coefficients for all factors. The intrinsic consistency coefficients for all factors were within acceptable limits. The correlation matrix shown in Table 1 indicates that all factors correlated with each other, with SA being significantly negatively correlated with the dimensions of job satisfaction (*p* < 0.001) and DA and ENFE being significantly positively correlated with the dimensions of job satisfaction.

**TABLE 1:** Descriptive statistics and correlations among variables.

	SA	DA	ENFE	IJS	EJS
Surface acting (SA)	(0.90)				
Deep acting (DA)	−0.35***	(0.82)			
Expression of naturally felt emotions (ENFE)	−0.40***	0.74***	(0.80)		
Intrinsic job satisfaction (IJS)	−0.53***	0.77***	0.80***	(0.91)	
Extrinsic job satisfaction (EJS)	−0.52***	0.71***	0.73***	0.90***	(0.92)
Mean	2.37	3.79	4.03	3.89	3.71
Standard deviation	0.88	0.76	0.82	0.72	0.78

***p < 0.001; Cronbach’ s α reliability coefficients in parentheses along the diagonal.

### Latent class enumeration

Selecting the best fitting profile solution is one of the important steps in LPA research. Referring to the recommendations of Spurk et al. (2020), we used a stepwise approach to determine the number of latent profiles that best characterized the data and sample, starting with an LPA with two profiles and successively adding profiles (Nylund et al., 2007). In each step, we examined the fit information criteria shown in Table 2 to determine the final number of profiles. We also considered theoretical coherence, discrimination, and profile size to determine the final number of profiles.

**TABLE 2:** Statistical criteria associated with latent class enumeration.

Model	K	LL	AIC	BIC	SABIC	Entropy	LMR (*p*)	BLRT (*p*)
2 classes	10	−1092.44	2204.87	2243.87	2212.15	0.94	0.004	<0.001
3 classes	14	−1019.40	2066.80	2121.40	2076.98	0.94	<0.001	<0.001
4 classes	18	−955.75	1947.51	2017.71	1960.60	0.93	0.257	<0.001
5 classes	22	−912.96	1869.92	1955.72	1885.92	0.96	0.006	<0.001

BLRT(*p*) = *p*-Value for the bootstrapped likelihood ratio test. LMR(*p*) = *p*-Value for the adjusted Lo-Mendell-Rubin-test.

All models converged fully during the estimation. This suggests that local maxima were not reached, and the best overall solution for the analysis was obtained. Table 2 shows the fit indexes of the tested models. The BIC value was lowest for the five-profile model, and the BLRT value demonstrated a better fit for the five-profile model compared to the models with three profiles and four profiles. Moreover, AIC values decreased with increasing numbers of profiles. Although the five-profile model had better fit indexes (AIC, BIC, and SABIC), the LMR-test supported the three-profile model since the four-profile model (*p* = 0.257) and five-profile model (*p* = 0.006) did not fit better than the three-profile model. We also considered entropy, which is a summary measure of the quality of classification in an LPA model. Values close to 1 indicate good classification accuracy, whereas values close to 0 indicate a lack of accuracy. The entropy of the models did not fall below the 0.80 criterion in this analysis.

Based on the model fit and entropy, we believed that a choice had to be made between the three-profile and the five-profile solution. Hence, we further inspected the interpretability of the profiles. Although the five-profile model showed better fit indexes, one of the profiles in the five-profile solution comprised a sample of only six teachers, which was lower than the cut-off of 25 cases recommended by Lubke and Neale (2006). Moreover, the profiles in the three-profile solution corresponded closely with the three profiles previously identified by Cheung and Lun (2015). Therefore, we selected three profiles as the final model.

**FIGURE 1:**
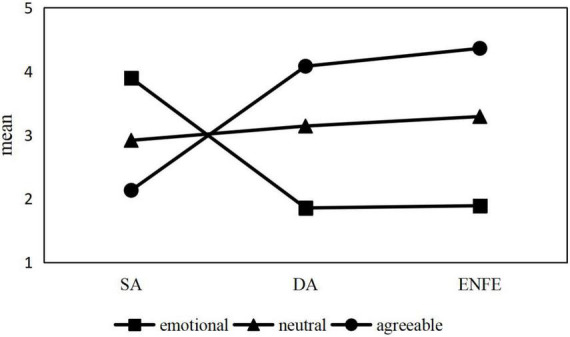
Mean scores for emotional labor items by latent class.

Figure 1 shows the mean values of the scores for each class of the EFL teachers’ emotional labor for SA, DA, and ENFE. Among the factors that influence teachers’ emotional labor, teachers’ personality traits are important individual factors that significantly influence their emotional labor (i.e., neuroticism tends to lead toward SA, and extraversion and agreeableness tend to lead toward DA and ENFE; Basim et al., 2013). Therefore, besides the scores for each strategy, we also referred to the Big-Five factors (i.e., extraversion, agreeableness, conscientiousness, openness, and neuroticism; Costa and McCrae, 1992), which scholars agree upon, and the way Chinese scholars classify Chinese personality traits (Xu et al., 2006) to assign names that captured the essence of the three classes.

The first class was labeled “emotional” (*n* = 26) because the teachers in this class scored highest on SA and lowest on both DA and ENFE. These findings revealed that teachers in this class had elevated levels of neuroticism and more superficial conditioning in their teaching work, but had difficulty cognitively understanding and accepting the rules of emotional presentation required for teaching. It has been shown that neuroticism predicts SA (Weiss and Cropanzano, 1996).

The second class was named “neutral” (*n* = 50) for those whose SA, DA, and ENFE scores were all moderate, indicating that this type of teacher had moderate intrinsic emotional involvement and moderate emotional expression. This naming followed Xu et al.’s (2006) classification of the personality structure of the Chinese Han, in which “Zhong” (moderation) and “He” (harmony) are the embodiment of an important traditional Chinese philosophical concept holding that emotions such as happiness, anger, sadness, and joy are impartial when they lie within the individual, and that these emotions are expressed in a moderate manner.

Finally, the third class was termed “agreeable” (*n* = 289) because the teachers in this class had relatively high levels of both DA and ENFE, indicating high levels of intrinsic emotional engagement and positive outward emotional expression.

### Comparison of emotional labor strategies across latent classes

In this study, we used a one-way ANOVA to explore differences in the use of emotional labor strategies across the three potential profile types. The results showed that there were significant differences in the use of all three emotional labor strategies. Table 3 presents the specific results of the one-way ANOVA. Overall, teachers with the first class (emotional) were likely to use SA but had the least use of DA and ENFE. Teachers with the third class (agreeable) used ENFE most frequently, followed by DA, but they had the lowest levels of SA.

**TABLE 3:** Descriptive statistics for each class and the total sample (*N* = 365).

Latent class	SA	DA	ENFE
Class 1 (emotional)	3.90 ± 0.74	1.85 ± 0.38	1.88 ± 0.42
Class 2 (neutral)	2.96 ± 0.59	3.13 ± 0.59	3.25 ± 0.44
Class 3 (agreeable)	2.13 ± 0.74	4.08 ± 0.40	4.36 ± 0.40
*F*	91.93***	391.31***	542.30***

***p < 0.001.

### Differences in job satisfaction among emotional labor profiles

In this study, a one-way ANOVA was conducted with IJS and EJS, as dependent variables and three potential profiles as independent variables as a way to explore the differences in job satisfaction among the three potential profiles of emotional labor (see Table 4).

**TABLE 4:** Results of ANOVA and *post hoc* comparisons on job satisfaction.

	IJS	EJS
Emotional	1.99 ± 0.48	1.84 ± 0.37
Neutral	3.30 ± 0.51	3.08 ± 0.48
Agreeable	4.17 ± 0.36	3.98 ± 0.51
*F*	438.50***	266.19***

***p < 0.001.

The results showed that for IJS, the main effect of the three potential profiles was significant: *F*(2,364) = 438.50, *p* < 0.001, η^2^_p_ = 0.71. To test exactly which two factors differed significantly, we conducted a *post hoc* comparison test based on a one-way ANOVA. The *post hoc* comparison revealed that the IJS of “agreeable” teachers was significantly higher than that of “neutral” and “emotional” teachers (*p* < 0.001), and the IJS of “neutral” teachers was significantly higher than that of “emotional” teachers (*p* < 0.001). For EJS, the main effect of the three potential profiles was also significant: *F*(2,364) = 266.19, *p* < 0.001, η^2^_p_ = 0.60. The *post hoc* comparison likewise confirmed that the EJS of “agreeable” teachers was significantly higher than that of “neutral” and “emotional” teachers (*p* < 0.001).

## Discussion

Our objective in the current study was to conceptualize high school EFL teachers’ emotional labor strategies into different behavioral patterns and then examine the relationship between these behavioral patterns and job satisfaction. We contribute to the literature by using person-centered analyses to examine the diverse types of EFL teachers represented by emotional labor strategy variables. The results of this research suggest that an LPA model with three distinct profiles of emotional labor provides an optimal solution. These findings support the idea that different profiles of emotional labor can be identified and replicated across cultures (Fouquereau et al., 2018).

### An application of the person-centered approach to English-as-a-foreign-language teachers’ emotional labor subgroups

A major theoretical contribution of this manuscript is that our profiles reconciled different views on how SA, DA, and ENFE relate to each other by demonstrating that different subgroups of EFL teachers can exhibit different combinations of strategies. Specifically, this study drew on Cheung and Lun (2015) conceptualization of the emotional labor construct. However, in contrast to the findings of previous studies, this study identified the emergence of emotional types (i.e., high SA, low DA, and low ENFE) in a class of high school EFL teachers. Teaching is a typical occupation for civil servants in China. Most teachers are recruited by the government and are expected to exhibit self-sacrifice and dedication, as well as to place the public interest first (Hofstede, 1980; Li and Wang, 2014). Teachers are strictly expected to act like service workers, treating parents and students as customers. Also, teaching in high school curriculum reform can produce feelings of guilt, disappointment, and exhaustion for EFL teachers who are unable to reconcile the conflicts between their felt dedication to student-centered learning and the unfeeling expectations imposed by a performance-oriented education system (Loh and Liew, 2016). When their feelings conflict with their image of the ideal teachers, high school teachers will display SA by pretending to be warm and caring to create positive emotional experiences for parents and students (Chiang, 2009; Brown et al., 2014).

In particular, we found a class indicating that SA, DA, and ENFE can coexist at comparable levels within (neutral) EFL teachers, consistent with the idea that emotion regulation strategies are positively correlated and can be used in tandem (Beal and Trougakos, 2013; Gabriel et al., 2015). This also reflects the critical role of Chinese cultural values in shaping and regulating teachers’ emotions (Zhang and Zhu, 2008). Some studies have proposed that emotional labor strategies are mutually exclusive, with the use of one strategy implying the non-use of another (Kruml and Geddes, 2000; Austin et al., 2008). In contrast, the findings of this study support the idea that teachers’ different emotional labor strategies coexist (Sutton, 2004; Zhang and Zhu, 2008). This suggests that high school EFL teachers have a better balance of emotion management.

We reasoned that two classes—agreeable and emotional—suggest that only one may be used primarily, perhaps at the expense of the other two, demonstrating a negative correlation between the strategies (Austin et al., 2008). By modeling the heterogeneity of Chinese EFL teachers, we show that these two theoretical perspectives can coexist across different subgroups of EFL teachers. Also, it is important to note that not only were emotional teachers specific in terms of emotional labor traits, but they were also few, representing only 7.12% of the sample. It is likely that this class would not have been detected if a variable-centered approach had been used, reflecting the advantage of using a person-centered approach in this study.

It is also interesting that the latent emotional labor profiles identified in this study did not have combined SA and ENFE, which is consistent with the finding of a significant negative association between these two strategies found in previous research (Diefendorff et al., 2005; Dewaele and Wu, 2021). This finding suggests that the potential profiles identified in this study are reasonable and meaningful—not merely random combinations of the three emotional labor strategies. Furthermore, the agreeable and emotional EFL teachers identified in this study revealed that there may also be a negative relationship between teachers’ emotional labor strategies. Accordingly, this manuscript hypothesizes that both positive and negative correlations may exist among SA, DA, and ENFE, only the groups differed, which is a beneficial supplement to previous variable-centered studies and provides a research direction for future related studies.

### Correlation between emotional labor subgroups and English-as-a-foreign-language teachers’ job satisfaction

An important aim of this research was to examine the correlations between emotional labor profiles and job satisfaction. We found that low levels of DA and ENFE were more detrimental to EFL teachers’ EJS when they simultaneously engaged in high levels of emotional SA. Based on the COR (Hobfoll, 1989), this result was mainly obtained because EFL teachers who adopted SA were extremely sensitive to working conditions and school demands, which increased their concerns about working conditions, salaries, and management rules. Specifically, English has a prominent place in China’s senior secondary education. In the demanding atmosphere surrounding English education, EFL teachers tend to suppress their negative emotions and display only positive ones despite the clash between what they hope to reveal and what they need to demonstrate (Cowie, 2011). EFL teachers consider it their moral responsibility to regulate their emotions and display appropriate emotions in a given teaching environment according to their professional teaching norms. Their emotional displays (e.g., pretending or hiding) consume a significant amount of psychological resources, ultimately resulting in a net loss of individual psychological resources and low job satisfaction (Ghanizadeh and Royaei, 2015).

In contrast to the emotional type, appropriate SA may be beneficial to EFL teachers when combined with high levels of DA and ENFE (neutral). In fact, neutral teachers had moderate levels of IJS and EJS, outperforming emotional teachers. This suggests that SA may be performed without causing harm to EFL teachers since teaching requires teachers to use elevated levels of DA and ENFE. A Previous study indicated that SA is harmful to Chinese teachers (e.g., Li and Wang, 2014). We suggest that this may have arisen because the two subgroups (emotional and neutral) that engaged in high SA were combined in the previous sample. It has been noted that personal and social identities influence how individuals evaluate situations, further moderating the relationships between emotional labor strategies and their outcomes, especially in terms of cognitive outcomes, such as teaching satisfaction (Shamir et al., 1993; Humphrey et al., 2015). From the perspective of high school EFL teachers in mainland China, their various types of emotional displays are relatively tolerable and do not necessarily lead to job dissatisfaction because the teachers generally accept greater power distance (Hargreaves, 2000; Fung and You, 2011) and have more authority to express intense emotions (both positive and negative) in the classroom (Huang et al., 2019), and use this method to capture students’ interest and maintain their attention. In this case, for teachers who revel in the artistry of pedagogical performances (Eisner, 1983; Liew, 2013), teaching can be an invigorating form of emotional labor.

Also, the use of other strategies, such as DA, reflects teachers’ identification with their job roles (the role demonstrates the transmission of rules through emotional labor). Noddings (1984) argued that an “ethic of care” is best demonstrated when teachers see the act of engaging in emotional labor as part of the development of a teaching identity and learn to internalize it. Thus, the simultaneous use of DA and ENFE may mitigate the internal and external emotional dysregulation caused by SA and reduce its negative impact on both IS and ES.

Although neutral teachers had higher IJS and EJS than emotional teachers, their job satisfaction was still lower than that of agreeable teachers. One interesting finding was that both agreeable and neutral teachers frequently used DA; the former also showed more ENFE, and consequently, their job satisfaction, especially their IJS, was the highest. First, it should be noted that EFL teachers who perform DA usually strongly agree with their schools’ goals, which facilitates more concern for the intrinsic values of education than external interests. Although these emotional displays can lead to dehumanizing consequences when used over time (Sammons et al., 2007), teachers still experience higher job satisfaction because positive work outcomes (e.g., job performance; Grandey, 2000) can help teachers to restore or regain psychological resources (Cheung et al., 2011). Thus, high school EFL teachers who use DA tend to believe that their high job satisfaction stems from the value of their schoolwork, which is also reflected in their high IJS.

Of course, as noted above, teachers’ prolong their use of DA to isolate their professional identities from their personal dimensions for extended periods, which contributes to the maintenance of their personal–professional well-being but actually presupposes individual personality fragmentation (Sammons et al., 2007). If teachers are unable to replenish their resources promptly, the resulting stress may lead to higher rates of burnout and lower job satisfaction (Hobfoll, 1989). We found that the (agreeable) class using more frequent ENFE alongside high levels of DA tended to report higher levels of IJS and EJS. This finding is in line with the findings of previous research (e.g., Cheung et al., 2018). One possible explanation is that teachers can show their professionalism and give students confidence by directly sharing their inner emotions with them, which is probably less costly than faking positive emotions (Dewaele and Wu, 2021). Adopting more ENFE can thus significantly improve EFL teachers’ sense of accomplishment, experience “emotional consonance” between their actions and emotions, and find their labors of love profoundly gratifying (Isenbarger and Zembylas, 2006; O’Connor, 2008), which in turn increases their job satisfaction, especially the IJS. It can be argued that ENFE, as an adaptive way of regulating emotions, is the only one that predicts teachers’ job satisfaction (Yin et al., 2013) and has a more positive effect than trying (DA) or pretending (SA) to feel an emotion (Akin et al., 2014; Huang et al., 2019).

### Implications

Several practical recommendations emerged from this research. Based on our results, school administrators may wish to ensure that EFL teachers use fairly low levels of SA or focus on DA and ENFE. From a selection standpoint, this may involve identifying agreeable or neutral teachers who are highly attuned to the emotional demands of FL teaching. This would be advantageous in the long term, as agreeable and neutral teachers exhibit some of the highest well-being and are likely to ensure their positive development in the workplace. If EFL teachers are in a class that exhibits low job satisfaction, such as the emotional teachers in this study, who exhibited the lowest IJS and EJS, administrators may need to consider whether to foster conditions that can buffer the effects of profile membership on teachers’ job satisfaction.

First, the findings of this study revealed that EFL teachers who tended to use SA exhibited the lowest EJS. The most essential aspect of the new curriculum reform in high schools is the change in the role and nature of teachers’ work. Every teacher is expected to be a developer and researcher of the curriculum, which is extremely challenging for teachers. Although the exogenous pressure of school effectiveness and assessment can stimulate the vitality of teachers, it also affects their emotions to a large degree, which can lead to teachers to choose to work with SA. Our findings highlight the significance and need of the institutional environment in guiding teachers’ emotional labor in a positive way. Based on this, administrators should respect the job characteristics of high school EFL teachers and foster a relatively flexible institutional environment so that the regulations and organizational expectations are consistent with teachers’ emotions, thus helping teachers develop positive work attitudes and enhance their job satisfaction.

Second, the significant positive impact of agreeable teachers on IJS and EJS highlights the importance of emotional displays that coincide with teachers’ feelings at school. It may be beneficial to provide EFL teachers with conditions for DA and ENFE. In this sense, the management of emotional labor is necessary. As Benesch (2018, 2020) argued, both emotional rules and emotional labor embody teachers’ agency, and teachers need a deep understanding of emotional rules to better use their emotional labor to resist organizational power. In the Chinese cultural context, a human-oriented organizational culture, including democratic decision-making, encouragement of pedagogical innovation, and support for professional development would be beneficial in stimulating EFL teachers’ endogenous motivation to carry out teaching and research, promoting the use of ENFE or DA, and thus contributing to teachers’ IJS.

### Limitations and suggestions for future research

To best weigh the contributions of the current study, some limitations of this study should be considered. The outcome variables selected for this study were at the individual level, and researchers have noted that research on outcome variables for emotional labor should explore the balance between organizations and individuals (Grandey and Gabriel, 2015). Therefore, whether the potential categories noted as beneficial can simultaneously benefit organizations (e.g., high performance or organizational climate) needs to be further explored. Moreover, the current study focused only on preliminary results for teachers’ emotional labor, but did not address the relationship between these profiles between teachers’ demographics (e.g., age or education) or teaching experience (e.g., years of teaching experience or title). Thus, future research should consider examining a broader range of factors influencing teachers’ emotional labor, such as at the individual level (e.g., personality traits or emotional intelligence) and at the organizational level (e.g., leadership style, perceived social support or workload).

Additionally, this research concentrated on the microsystem of classroom teaching. As mentioned earlier, teachers are often weighed down by the impact of reforms on their teaching practices and philosophies. For this reason, future research should focus on the frequency, intensity, and persistence of the emotional labor that EFL teachers expend at various levels of the reform ecosystem, take a macro perspective, and/or use a longitudinal and reciprocal research model (e.g., group discussions, interviews, and/or observations; Chen, 2019) to comprehensively examine the connotations and dynamic processes of teachers’ emotional labor. Finally, although the current study provides a useful addition to studies on EFL teachers’ emotional labor in China, it was not possible to determine whether the potential categories identified in the study are unique to teachers in Chinese cultural contexts. Future research should use samples of teachers from different ethnic and cultural backgrounds to account for the specificity of teachers’ affective experiences in different regional or cultural contexts.

## Conclusion

In conclusion, this study suggests that EFL teachers who are able to use a combination of DA and ENFE may report higher levels of IJS and EJS. In contrast, high frequencies of SA always seem to be detrimental to teacher’s job satisfaction, especially EJS. We encourage researchers and practitioners to further evaluate the use of person-centered approaches, such as LPA, as applied to emotional labor strategy variables to examine the nature of the diverse types of EFL teachers represented by the domains of emotional labor. Such a pursuit will not only facilitate alignment between the emotional labor strategies model and analytical methods but will also reveal whether and how different emotional labor profiles have interesting associations with outcomes that might otherwise be overlooked by focusing exclusively on variable-centered approaches.

## Data availability statement

The original contributions presented in this study are included in the article/supplementary material, further inquiries can be directed to the corresponding author.

## Ethics statement

The studies involving human participants were reviewed and approved by Teacher Development Committee, College of Foreign Studies, Guangxi Normal University. The patients/participants provided their written informed consent to participate in this study. Written informed consent was obtained from the individual(s) for the publication of any potentially identifiable images or data included in this article.

## Author contributions

SZ contributed to conception and design of the study and wrote the first draft of the manuscript. MZ organized the database and performed the statistical analysis. Both authors contributed to manuscript revision, read, and approved the submitted version.

## Footnotes

1 Reform and opening up is the policy of intrinsic reform and extrinsic opening up that China has been implementing since 1978. Foreign language education in China has also been flourishing since then.

2 Gaokao, the National College Entrance Examination, is an academic testing event held annually in China and joined by millions of participants, a majority of whom are high school students.

## Appendix A

### Emotional labor scale (by Diefendorff et al., 2005)

1. I put on an act in order to deal with students or colleagues in an appropriate way.
2. The emotions I express to students or colleagues are genuine.
3. I fake a good mood when interacting with students or colleagues.
4. I try to actually experience the emotions that I must show to students or colleagues.
5. I put on a “show” or “performance” when interacting with students or colleagues.
6. I just pretend to have the emotions I need to display for my job.
7. I make an effort to actually feel the emotions that I need to display toward students or colleagues.
8. I put on a “mask” in order to display the emotions I need for the job.
9. I work hard to feel the emotions that I need to show to students or colleagues.
10. I show feelings to students or colleagues that are different from what I feel inside.
11. I work at developing the feelings inside of me that I need to show to students or colleagues.
12. The emotions I show students or colleagues come naturally.
13. The emotions I show students or colleagues match what I spontaneously feel.
14. I fake the emotions I show when dealing with students or colleagues. (Page)

### Job satisfaction (by Weiss et al., 1967)

1. Being able to keep busy all the time.
2. The chance to work alone on the job.
3. The chance to do different things from time to time.
4. The chance to be “somebody” in the community.
5. The way my leader handless teachers.
6. The competence my supervisor in making decision.
7. Being able to do things that don’t go against my conscience.
8. The way my job providers for steady employment.
9. The chance to do things with other people.
10. The chance to tell people what to do.
11. The chance to do something that makes use of my abilities.
12. The way school policies are put into practice.
13. My pay and the amount of work I do.
14. The chances for advancement on this job.
15. The freedom to use my own judgment.
16. The chance to try me own methods of doing the job.
17. The working conditions.
18. The way my co-workers get along with each other.
19. The praise I get for doing a good job.
20. The feeling of accomplishment I get from the job.
